# Tn*6553,* a Tn*7*-family transposon encoding putative iron uptake functions found in *Acinetobacter*

**DOI:** 10.1007/s00203-022-03291-0

**Published:** 2022-10-26

**Authors:** Mehrad Hamidian

**Affiliations:** Australian Institute for Microbiology and Infection, University of Technology Sydney, Ultimo, NSW 2007 Australia

**Keywords:** *Acinetobacter baumannii*, Tn*6553*, Tn*7* family transposon, Iron acquisition

## Abstract

*Acinetobacter baumannii* is an opportunistic pathogen that has become difficult to eradicate mainly because of its high level of antibiotic resistance. Other features that contribute to this organism's success are the ability to compete for nutrients and iron. Recently, several novel Tn*7*-family transposons that encode synthesis and transport of siderophore and iron uptake systems were characterised. Here, another Tn*7*-type transposon (named Tn*6553*) is described. Tn*6553* contains a set of iron utilisation genes with a transposition module related to Tn*7*. Tn*7-*family transposons that carry iron uptake systems facilitate the spread of these functions in *Acinetobacter* strains. Given that Tn*7* is known to transpose efficiently into its preferred target site, finding siderophore functions on Tn*7* family transposons is important in the context of dissemination of virulence genes amongst *Acinetobacter* strains.

## Introduction

*Acinetobacter baumannii* has proven to be a successful pathogen mainly due to high levels of antibiotic resistance, producing biofilm and the ability to compete for iron under iron-limiting conditions (Zimbler et al. 2009; Zarrilli et al. 2013; Harding et al. 2018). Their ability to compete for micronutrients required for growth, e.g. iron, contributes to their success allowing this organism to become a successful pathogen (Mortensen and Skaar 2012; Harding et al. 2018). Recently, we described a set of large novel transposons that carry genes for synthesising and transporting siderophores in several unrelated strains (Hamidian and Hall 2021). We showed that these transposons (Tn*6171*, Tn*6552* and their variants) contain genes encoding transposition proteins related to those in Tn*7* (Hamidian and Hall 2021) and that they can target the *glmS* gene, which is the preferred Tn*7* target site (Craig 1991, 1996). Similar to Tn*7* (Craig 1991, 1996)*,* Tn*6171*, Tn*6552* and their variants are flanked by five (*n* = 5) bp target site duplications (TSD) (Hamidian and Hall 2021). Here, the properties of yet another Tn*7*-family transposon designated Tn*6553* that encodes iron uptake functions are described. This adds another mobile genetic element to the growing list of Tn*7-*family transposons that can disseminate important functions in *Acinetobacter* strains.

## Methods

### Sequence data used in this study

Complete genome sequences of the *Acinetobacter* strains, *Acinetobacter soli* strain GFJ2 and *A. baumannii* DS002 GenBank accession numbers CP027704.2 and CP016896, respectively, were used in this study to examine the structure of Tn*6553* and its variant Tn*6553*-v1.

### Sequence annotation and analysis

Standalone BLAST (available at https://www.ncbi.nlm.nih.gov/books/NBK52640) functions, including BLASTn, BLASTp and tBLASTn, were used for sequence analysis as previously described (Altschul et al. 1990). All coding regions of Tn*6553* were found using the NCBI’s Orf Finder program available at https://www.ncbi.nlm.nih.gov/orffinder. Gene features of transposons including all protein-coding regions were annotated manually using both Pfam (http://pfam.xfam.org/), UniProt (https://www.uniprot.org) and BLASTP (http://blast.ncbi.nlm.nih.gov/Blast.cgi) searches as described previously (Altschul et al. 1990; Finn et al. 2014, 2017). The IS-Finder database (https://www-is.biotoul.fr/) was used to identify insertion sequences (IS). Conserved sequences were determined using the WebLogo software available at https://weblogo.berkeley.edu/logo.cgi.

## Results and discussion

**Fig. 1:**
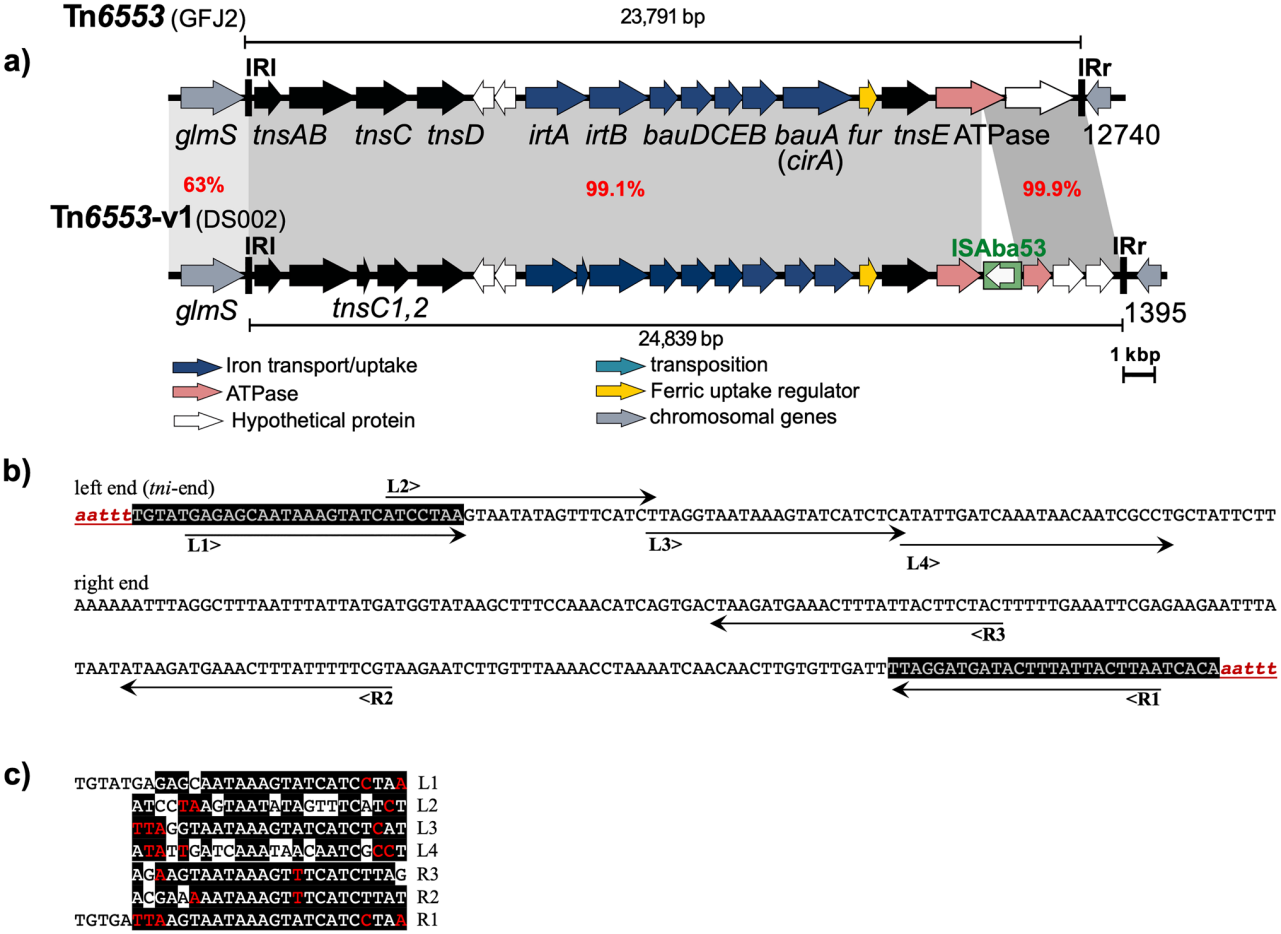
Genetic structures of Tn*6553* and Tn*6553*-v1 **a**, Inverted repeats (IR) of Tn*6553* **b** and sequence conservation in transposon binding sites of Tn*6553* **c**. In **a**, horizontal arrows indicate the direction and orientation of genes. Transposition genes are coloured black, iron uptake systems are dark blue, chromosomal genes are grey and white indicating hypothetical proteins. **b**, IR and transposon binding sites of Tn*6553.* Target site duplications are in lower case and red (5 bp). The left and right end of Tn*6553* is shown using uppercase letters with IRs grey on black. Horizontal arrows below and above the sequence lines (L1-4 and R1-3) are transposon binding sites, TBSs. **c**, sequence conservation of IRs and TBSs. Conserved sequences are shown using white or red letters on black (on the left) and a WebLogo (on the right) generated using the software available at https://weblogo.berkeley.edu/logo.cgi

*A. baumannii* strains have become successful opportunistic superbugs that have become difficult to treat because of the acquisition of genes conferring resistance to a wide range of antibiotics including carbapenems (Hamidian and Nigro 2019). Their success is also partly due to their ability to compete for micronutrients including iron (Mortensen and Skaar 2012; Harding et al. 2018). We previously described two Tn*7*-family transposons that carry iron uptake and siderophore functions that could increase the iron uptake capacity of strains that carry them by supplying the genes required for synthesis and uptake of the fimsbactin group of siderophores (Hamidian and Hall 2021). Here, searches of the complete genomes of *Acinetobacter* strains in GenBank non-redundant database using low stringency BLASTN and sequence of the boundaries of Tn*6171* uncovered yet another Tn*7* family transposon type, encoding a predicted iron uptake system. This transposon is located downstream of the chromosomal *glmS* gene of the *Acinetobacter soli* strain GFJ2 (GenBank accession number CP016896) and is flanked by five (*n* = 5) bp TSD (Fig. 1a, b). This transposon is novel and hence was herein named Tn*6553*. Tn*6553* is a 23791 bp Tn*7* family transposon, bounded by 28 bp imperfect inverted repeats, and like Tn*6171* and Tn*6552*, further transposon binding sites for TnsB were detected within 200 bp of the Tn boundaries (Fig. 1b, c). Tn*6553* includes a complete set of genes (*tnsABCDE*) encoding transposition proteins with 41–48% aa identities to TnsABCD and 26% identity to TnsE encoded by Tn*7* (and with 32–43% aa compared to TnsABCDE in Tn*6171*). However, the *tns* module is interrupted by the iron acquisition segment located between the *tnsD* and *tnsE* genes.

Tn*6553* encodes a set of functions required for iron uptake. It contains several genes such as *irtAB, bauDCEBA* and *fur*, which mainly encode proteins predicted to be involved in ferrichrome uptake and transmembrane transport (Fig. 1a), which are distantly related to *Escherichia coli* ferrichrome-iron receptor (Coulton et al. 1983). The *irtAB* genes encode putative ABC-type multidrug transport systems (ATPase and permease component) belonging to the MdiB superfamily (Protein family accession number COG1132). The *bauDCE* genes encode putative iron chelate uptake ABC transporter family permease subunit (ferric acinetobactin ABC transporter), while *bauB* predicted to encode a siderophore-binding periplasmic lipoprotein and *bauA* a TonB-dependent siderophore protein (Fig. 1a). Tn*6553* also carries a *fur* gene encoding an Fe2 + (or Zn2 +) uptake transcriptional regulation protein (Protein family accession number COG0732), which is in fact a putative master transcriptional regulator of the iron-responsive genes. Notably, the iron uptake proteins encoded by Tn*6553* are distantly related to the siderophore systems (with amino acid identities ranging from 20–30%) that we recently described in *A. baumannii* in different Tn*7-*family transposons (Hamidian and Hall 2021). However, whether this operon functions as a siderophore system remains to be experimentally examined.

A recent study reported that *A. baumannii* DS002 (GenBank accession number CP027704.2) contains a novel siderophore system present in a genomic island that encodes 30 genes/orfs including four (*n* = 4) genes encoding putative transposases TnsBC1C2E, 13 gene encoding putative iron acquisition functions, and the 13 reading frames coding for proteins of unknown functions (Yakkala et al. 2019). However, detailed sequence analysis showed that this iron uptake system is in a variant of Tn*6553*, here called Tn*6553*-v1 (Fig. 1a). Tn*6553*-v1 is 24,839 bp in size. It encodes 23 open reading frames, including transposition functions, iron acquisition, DNA metabolism and four (*n* = 4) hypothetical proteins. Tn*6553*-v1 is 1047 bp longer in size than Tn*6553* due to the insertion of a novel 1039 bp IS*5* family insertion sequence, named ISAba53 (Fig. 1a), and it’s eight (*n* = 8) bp TSD. In addition to the presence of a copy of ISAba53 (a novel IS*5* family insertion sequence that is 85% identical to ISAha1) in Tn*6553*-v1, it also differed from Tn*6553* by 17 bp and six (*n* = 6) bp gaps across the remaining segments. Notably, the *tnsC* gene is split into two (*n* = 2) reading frames (Fig. 1a), likely due to sequencing errors. The *itrB* gene was also divided into two (*n* = 2) frames, probably due to sequencing or assembly errors. However, these could not be verified here as the strain was not accessible. Together, the Tn*7* family transposon found in DS002 (Tn*6553*-v1) is an ISAba53 interrupted variant of Tn*6553* (Tn*6553*::ISAba53), indicating that Tn*6553* is more ancestral and that *A. soli* might be the source. This is consistent with both isolates recovered and evolved in the same environmental niche, soil. Differences in TniABCDE of Tn*6553* compared to those in Tn*7*, Tn*6171* and Tn*6552* (~ 48–52% aa identity) indicate that these Tn*7* family transposons belong to a very diverse family.

**Fig. 2:**
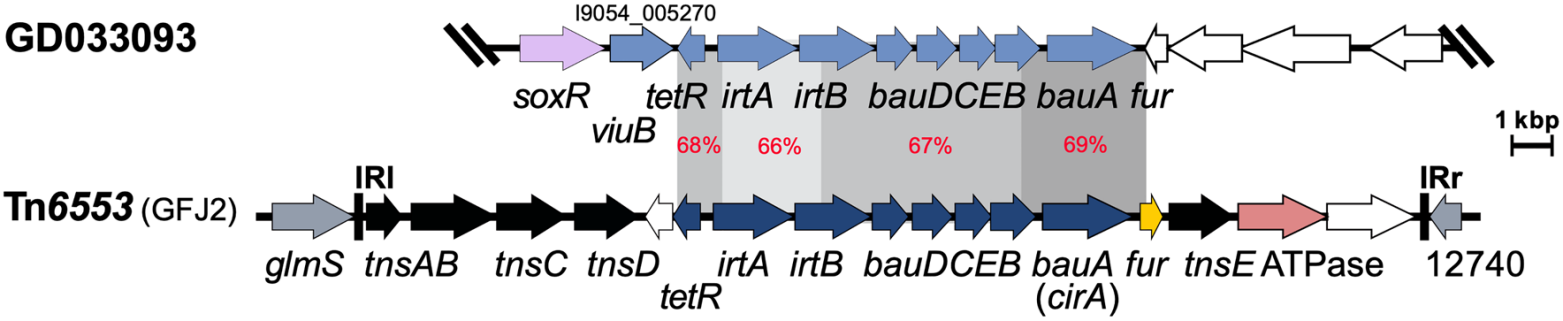
Comparison of the putative iron uptake region of Tn*6553* with the corresponding region in *Acinetobacter bereziniae* strain GD03393. Horizontal arrows indicate the direction and orientation of genes. Arrows coloured different shades of blue represent putative iron uptake genes. Transposition genes of Tn*6553* are coloured black. Scale bar is shown. Regions with significant homology are shown using shades of grey with their DNA identities indicates in red. Figure is drawn to scale from GenBank accession numbers CP092085 (GD03393 chromosome) and CP016896 (GFJ2 chromosome)

Searching the GenBank non-redundant database; however, a region distantly related to the putative iron uptake region of Tn*6553* (with approx. 66–69% DNA identity) was found in several *Acinetobacter* strains, namely *Acinetobacter* sp. Tol 5 DNA, *Acinetobacter bereziniae* strain GD03255, *Acinetobacter bereziniae* strain GD03393 (shown as a representative in Fig. 2), *A. bereziniae* strain GD03185, *A. bereziniae* strain XH901 and *Acinetobacter guillouiae* NBRC 110,550 (GenBank accession numbers, CP092085 AP024708.1, CP092083.1, CP066119.1, CP018259.1 and AP014630.1) (Fig. 2). This included an approximately 8 kb segment starting from the 5’-end of the *tetR* gene extending to ~ 100 bp downstream of the *bauA* gene (Fig. 2). Notably, this putative iron uptake region included an additional gene, namely *viuB* (locus id_ I9054_005270 in GenBank accession number CP092085)*,* encoding a NADPH-dependent ferric siderophore reductase. This gene is missing in Tn*6553.* These *Acinetobacter* strains belong to different species (*bereziniae* and *guillouiae*) and given that they are recovered from diverse clinical and environmental samples in different geographical regions indicating that this putative iron uptake system is not restricted to a specific species, region, or niche. However, given that no putative iron uptake region with significant homology was found outside the *Acinetobacter* genus also suggests that *Acinetobacter* is the origin.

This work describes yet another novel Tn7 family transposon type that encodes a predicted iron uptake system. Despite evolutionary differences these transposons share the same properties and are found in the same chromosomal position as Tn7. Finding siderophore functions on Tn7 family transposons in an important finding in the context of dissemination of virulence genes amongst *Acinetobacter* strains given that Tn*7* is known to transpose very efficiently into its preferred target site. The presence of siderophore/iron uptake systems on Tn*7-*family transposons facilitates the spread of these functions providing further evidence on strategies that *Acinetobacter* strains use to rapidly evolve, compete, and adapt under extreme conditions. None of the transposons that carry iron uptake/siderophore function characterised to date (Tn*6171*, Tn*6552* and Tn*6553*) have been found outside the *Acinetobacter* genus, however, as more genomes are sequenced additional related transposon might likely be found in this genus and maybe in other bacterial genera.

## Data Availability

All genome sequence data analysed during this study are publicly available in GenBank and cited in the article.
